# Synthesis and characterization of a novel eco-friendly corrosion inhibition for mild steel in 1 M hydrochloric acid

**DOI:** 10.1038/srep19890

**Published:** 2016-01-22

**Authors:** Ahmed A. Al-Amiery, Fatin A. Binti Kassim, Abdul Amir H. Kadhum, Abu Bakar Mohamad

**Affiliations:** 1 Department of Chemical and Process Engineering, Faculty of Engineering and Built Environment, Universiti Kebangsaan Malaysia, Bangi, Selangor 43600, Malaysia; 2 Environmental Research Center, University of Technology, Baghdad 10066, Iraq

## Abstract

The acid corrosion inhibition process of mild steel in 1 M HCl by azelaic acid dihydrazide has been investigated using electrochemical impedance spectroscopy (EIS), potentiodynamic polarization, open circuit potential (OCP) and electrochemical frequency modulation (EFM). Azelaic acid dihydrazide was synthesized, and its chemical structure was elucidated and confirmed using spectroscopic techniques (infrared, nuclear magnetic resonance and mass spectroscopy). Potentiodynamic polarization studies indicate that azelaic acid dihydrazide is a mixed-type inhibitor. The inhibition efficiency increases with increased inhibitor concentration and reaches its maximum of 93% at 5 × 10^−3^ M. The adsorption of the inhibitor on a mild steel surface obeys Langmuir’s adsorption isotherm. The effect of temperature on corrosion behavior in the presence of 5 × 10^−3^ M inhibitor was studied in the temperature range of 30–60 °C. The results indicated that inhibition efficiencies were enhanced with an increase in concentration of inhibitor and decreased with a rise in temperature. To inspect the surface morphology of inhibitor film on the mild steel surface, scanning electron microscopy (SEM) was used before and after immersion in 1.0 M HCl.

Mild steel is a widely used constructional material in many industries due to its acceptable mechanical properties and low cost^1^. The use of organic compounds to inhibit corrosion of mild steel and iron has assumed great significance due to the ability of these compounds to prevent corrosion in various corrosive environments^2^,^3^. A variety of organic compounds have been reported to be effective as corrosion inhibitors during acidization in industrial cleaning processes^4^,^5^. Organic additives prevent the adsorption of chloride ions and/or the formation of a more resistant oxide film on the metal surface^6^. The inhibition efficiency of these compounds depends mainly on the structure and nature of the adsorbed layer on the metal surface^7^,^8^,^9^,^10^,^11^,^12^,^13^,^14^,^15^,^16^,^17^,^18^. Organic inhibitors generally have heteroatoms, O, N, S and P, that are found to have higher basicity and electron density and thus act as corrosion inhibitors. O, N, S and P are active centers for the process of adsorption on metal surfaces. The inhibition efficiency should follow the sequence O < N < S < P. The use of organic compounds containing these atoms, especially nitrogen, to reduce corrosion attack on steel has been studied in some detail^19^. The availability of non-bonded (lone pair) and p-electrons in inhibitor molecules facilitates electron transfer from the inhibitor to the metal. A coordinate covalent bond, involving transfer of electrons from inhibitor to the metal surface, may be formed. The strength of the chemisorption bond depends upon the electron density of the donor atom in the functional group and also the polarizability of the group^20^. The electron density in the metal at the point of attachment changes, resulting in retardation of the cathodic or anodic reactions^21^. The effectiveness of the organic inhibitors depends on their adsorption rates and covering capabilities on metal surfaces. Many sources have determined that adsorption depends on the molecular structure and surface charge of the metal and type of electrolyte. Inhibitors adsorbed by a metal surface immersed in an aqueous phase replace water molecules adsorbed by the surface. Electrostatic interactions between inhibitor molecules and a metal are prominent during this inhibition action^22^. Adsorption depends mainly on certain physicochemical properties of the inhibitor group, such as electron density at the donor atom and orbital character and electronic structure of the molecule^23^. The adsorption of organic molecules at the metal/solution interface is of great interest in surface science and can markedly change the corrosion resistance of the metal^24^. It is generally accepted that the first step in the adsorption of an organic inhibitor on a metal surface usually involves replacement of one or more water molecules adsorbed at the metal surface^25^. The inhibitor may then combine with freshly generated Fe2+ ions on the steel surface, forming metal inhibitor complexes^26^,^27^. The organic inhibitors function through adsorption on the metal surface blocking the active sites by displacing water molecules and forming a compact barrier film to decrease the corrosion rate^28^. The adsorption of inhibitors on the metal/solution interface is influenced by: (i) the nature and surface charge of the metal; (ii) the type of aggressive electrolyte; and (iii) the chemical structure of the inhibitors^29^. Inhibitors act through a process of surface adsorption, so the efficiency of a given inhibitor depends on the characteristics of the environment in which it acts, the nature of the metal surface and electrochemical potential at the interface^30^,^31^. Inhibition efficiency is attributed to a combination of a moderation of film pH, reduction of chloride activity and concomitant release of inhibitor anions^32^,^33^,^34^. Acid solutions are widely used in industrial applications, such as acid pickling, industrial acid cleaning, acid descaling and oil well acidizing. Because of the general aggressiveness of acid solutions, inhibitors are commonly used to reduce corrosive attack on metallic materials^35^. The inclination towards eco-friendly corrosion inhibitor development intersects with several goals of pharmaceutical research, one of which is to discover or develop molecules with some desired biological activity. Efforts to attain this goal are strongly driven by the notion of molecular similarity because, in general, similar molecules tend to behave similarly^36^,^37^. In this study, azelaic acid derivative as a green inhibitor, namely azelaic acid dihydrazide, is synthesized, and its chemical structure is elucidated and confirmed using spectroscopic techniques. The inhibitory effect of azelaic acid dihydrazide on the corrosion of mild steel in 1.0 M HCl is investigated using various electrochemical measurements. Surface analyses are performed on the corroded surface using scanning electronic microscopy (SEM).

## Experimental

### Synthesis of corrosion inhibitor

All chemicals used in this synthesis were of reagent grade (supplied by Sigma-Aldrich, Selangor, Malaysia) and were used as received without further purification. The purity of the compounds was checked by thin-layer chromatography (TLC) on silica gel G plates with benzene, ethyl acetate, methanol 40:30:30 (v/v) or toluene: acetone 75:25 (v/v) as the mobile phase; the spots were located under UV light at 254 and 365 nm. Fourier transform infrared (FT-IR) spectra were recorded using a Thermo Scientific Nicolate 6700 FT-IR Spectrometer (Thermo Fisher Scientific, Waltham, MA, USA). Nuclear magnetic resonance (NMR) spectra were recorded using an AVANCE III 600 MHz spectrometer (Bruker, Billerica, MA, USA). The molecule fragments were calculated by using a GC-FID and GC-MS 7890 A system supplied by Agilent Technologies (Source Type ESI, Ion Polarity Positive, Set Capillary 4500 V, Set Dry Heater 180 °C, Set End Plate Offset -500 V, Set Dry Gas 5.0 l/min, Scan Begin 100 m/z, Scan End 1000 m/z, Set Collision Cell RF 250.0 Vpp).

### Azelaic acid ester

Azelaic acid ester can be synthesized according to the procedure reported by Charnock *et al.*^38^.

### Azelaic acid dihydrazide

A solution of azelaic acid ester (10 mmol) was refluxed with hydrazine hydrate (20 mmol) for 6 h. After concentrating the reaction mixture, a solid mass separated out and recrystallized using ethanol, yield 93%. Purity of the synthesized corrosion inhibitor was confirmed by thin layer chromatography (TLC). The TLC plates (20 × 10 cm) were recoated silica gel on aluminum 60F–254, with a stationary phase thickness of approximately 0.5 mm. Five microliters of each test solution was applied on each plate. The TLC plate was placed in a saturated chromatographic tank containing an ethyl acetate-methanol-acetone (25:25:50) solvent system. IR: 3313.8, 3289.5, 3199.6 and 3145.0 cm^−1^ (NHNH_2_), 3047.0 cm^−1^ (O=C−CH_2_−; aliphatic α-methylene groups), 2922.4 cm^−1^ (O=C−CH_2_−CH_2_−; aliphatic β-methylene groups), 2849.0 cm^−1^ (−CH_2_−; methylene groups) and 1632.2 cm^−1^ (C=O, amide). m/z: [217.16 (C_9_H_20_N_4_O_2_), 186.14 (C_9_H_18_N_2_O_2_), 156.12 (C_9_H_16_O_2_). 1H-NMR (CDCl_3_): δ 1.207 (m, 2H, for –CH_2_) and δ 1.412 (m, 2H, for CH_2_), δ 1.938 (m, 2H, for −CH_2_) and δ 8.882 (s, 2H, NH_2_). 13C-NMR (CDCl3): δ 172.14 (C=O); 28.98, 29.54, 33.60, 33.86, 34.13, 39.50, 39.64 (CH_2_).

### *Electrochemical measurements*

Mild steel specimens obtained from the Metal Samples Company were used as working electrodes throughout this study, each with an active surface area of 4.5 cm^2^. The composition (wt%) of the mild steel was as follows: Fe, 99.21; C, 0.21; Si, 0.38; P, 0.09; S, 0.05; Mn, 0.05; and Al, 0.01. The specimens were cleaned according to ASTM standard procedure G1-03^39^. Measurements were conducted in aerated, non-stirred 1.0 M HCl solutions containing different concentrations of azelaic acid dihydrazide as an inhibitor. Electrochemical measurements were performed at the steady-state corrosion potential using a Gamry water-jacketed glass cell. The cell contained three electrodes, working, counter and reference electrodes, which were composed of mild steel, a graphite bar and a saturated calomel electrode (SCE), respectively. The measurements were performed using the Gamry Instrument Potentiostat/Galvanostat/ZRA (REF 600) model (Gamry, Warminster, PA, USA). DC105 and EIS300 software by Gamry were used to perform the corrosion potential, potentiodynamic polarization, electrochemical impedance spectroscopy (EIS) and electrochemical frequency modulation (EFM) measurements. The potentiodynamic polarization curves were swept from −0.2 to +0.2 VSCE over the corrosion potential at a scan rate of 0.5 mV·s^−1^. EIS measurements were performed using the AC signals of the 5 mV peak-to-peak amplitude at the corrosion potential in the frequency range of 100 KHz to 0.1 Hz. All impedance data were fit to appropriate equivalent circuits (ECs) using the Gamry Echem Analyst software. EFM measurements were carried out at 0.1 Hz base frequency with applied AC potential of 10 mV for 20 cycles. The electrochemical measurements began to be collected approximately 30 min after the working electrode was immersed in the solution to allow the steady-state potential to stabilize. Each measurement was repeated three times, and only the average values were reported to verify reproducibility of the experiments.

## Results and Discussion

### Synthesis

**Figure 1:**
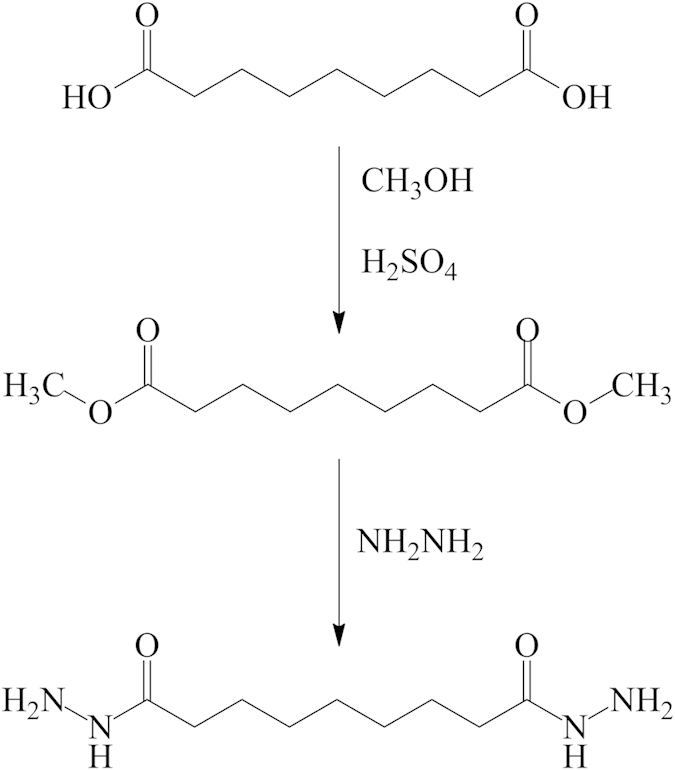
Chemical synthesis of azelaic acid dihydrazide.

To synthesize azelaic acid dihydrazide as a corrosion inhibitor, the reaction sequence outlined in Fig. 1 was followed, starting from commercially available azelaic acid.

**Figure 2:**
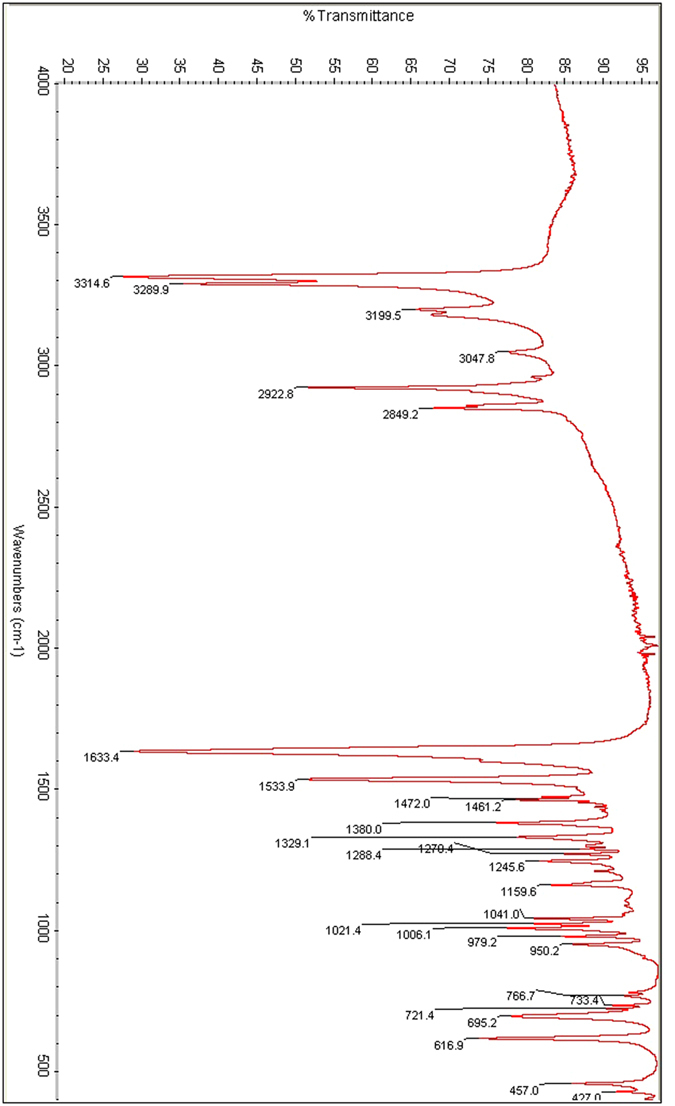
FT-IR spectrum for the corrosion inhibitor.

The synthesis was carried out by refluxing azelaic acid with methanol in the presence of sulfuric acid, followed by reaction with hydrazine hydrate. The molecular weight of the synthesized corrosion inhibitor is 216.16, which is calculated based on the molecular formula (C_9_H_20_N_4_O_2_) and supported *via* mass spectrometry. Azelaic acid dihydrazide can be dissolved in acetone, dichloromethane, dimethylformamide, dimethylsulfoxide, ethanol or methanol solutions. The FT-IR spectrum of this compound shows absorption bands at 3314, 3289 and 3199 cm^−1^ for hydrazide NH-NH_2_ and carbonyl stretching at 1633 cm^−1^. The bands at 2849 and 2922 cm^−1^ are from C-H aliphatic (Fig. 2).

**Figure 3:**
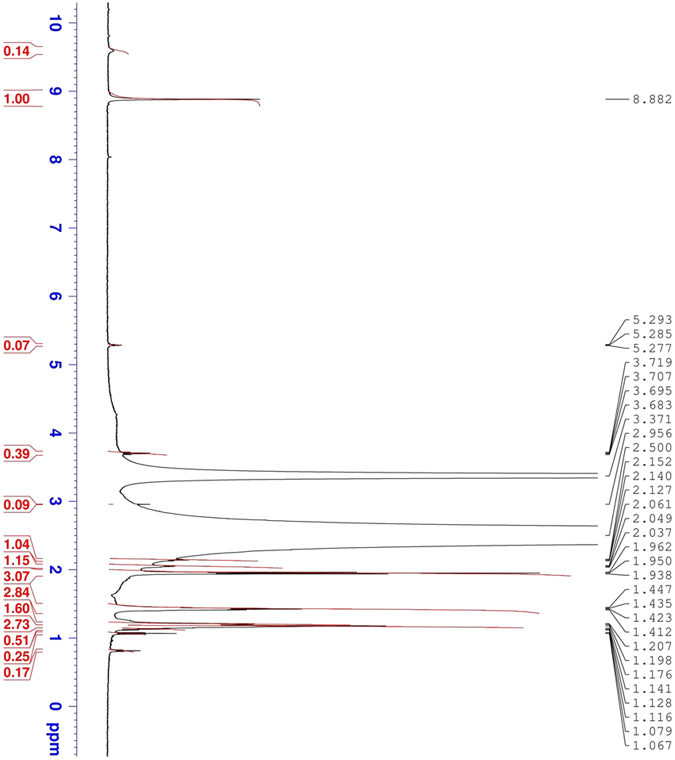
Proton-NMR spectrum for the corrosion inhibitor.

The ^1^H-NMR spectrum exhibits a singlet at δ 8.882 ppm due to the NHNH_2_ protons and also at δ 1.207 (m, 2H, for −CH_2_), δ 1.412 (m, 2H, for CH_2_) and δ 1.938 ppm (m, 2H, for −CH_2_), as shown in Fig. 3.

**Figure 4:**
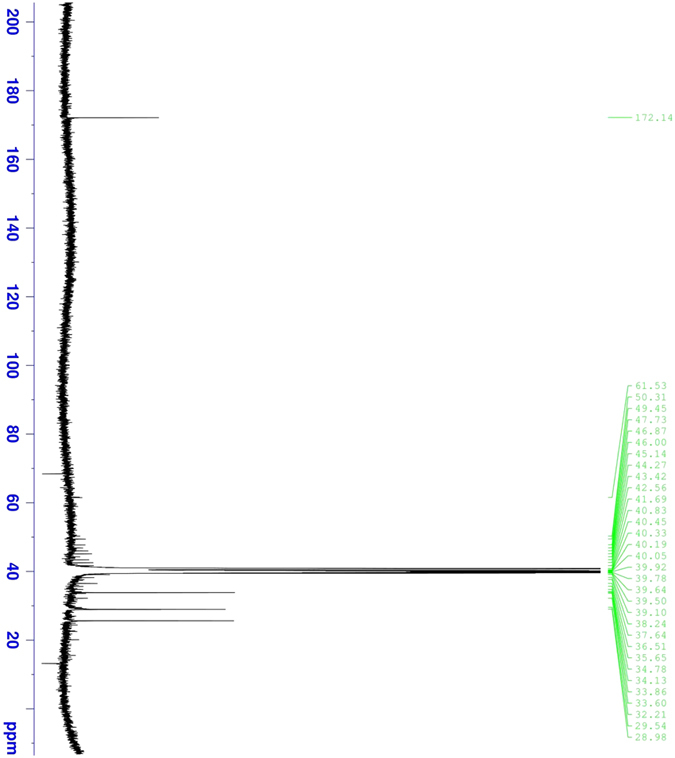
Carbon-NMR spectrum for the corrosion inhibitor.

From ^13^C-NMR, a band appears at 172.14 ppm due to the carbonyl group, and bands at 28.98, 29.54, 33.60, 33.86, 34.13, 39.50 and 39.64 ppm are from carbon atoms in the methylene groups (Fig. 4).

**Figure 5:**
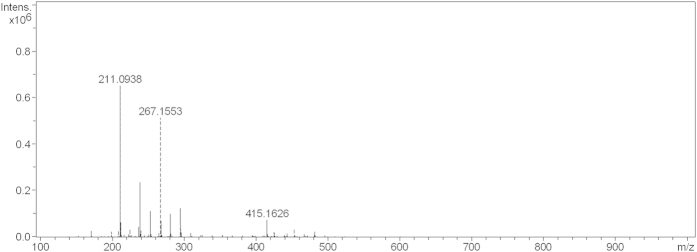
Mass spectrum for the corrosion inhibitor.

Mass spectrometry is a useful characterization method to confirm the structure of a compound. As shown in Fig. 5, m/z 217 indicates C-N bond cleavage to yield a carbonyl compound, m/z 186 represents cleavage of the N-C bond of a carbonyl compound, and m/z 156 corresponds to a di-carbonyl compound.

### Electrochemical results

#### Electrochemical impedance spectroscopy (EIS)

**Figure 6:**
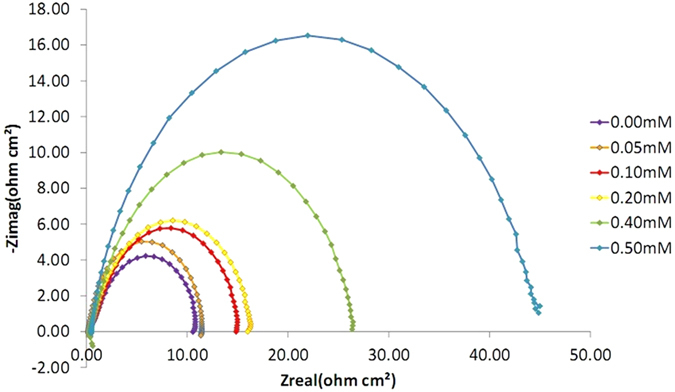
Nyquist plots for mild steel in 1.0 M HCl with various concentrations of azelaic acid dihydrazide at 30 °C.

**Figure 7:**
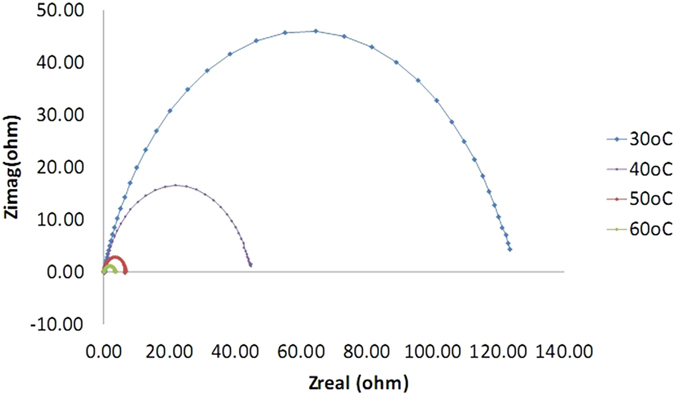
Nyquist plots for mild steel in 1.0 M HCl with 0.5 mM azelaic acid dihydrazide at various temperatures.

**Figure 8:**
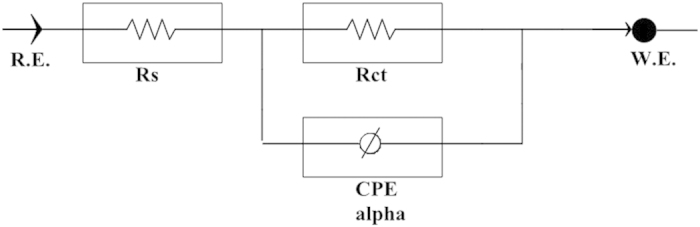
Equivalent model used to fit impedance data for mild steel in 1.0 M HCl in the absence and presence of azelaic acid dihydrazide.

The experimental results obtained from EIS measurements for the corrosion of mild steel in the absence and presence of the inhibitor at 30, 40, 50 and 60 °C are summarized in Tables 1 and 2. The impedance spectra for the mild steel samples in 1.0 M HCl in the absence of corrosion inhibitor (azelaic acid dihydrazide) or in the presence of various concentrations of corrosion inhibitor at 30 °C are presented as Nyquist plots in Fig. 6. A considerable increase in the total impedance is observed with the addition of corrosion inhibitor. As shown in Fig. 6, the impedance response of mild steel is significantly altered after the addition of inhibitor to the corrosive solution. This result can be attributed to an increase in the substrate impedance with increased inhibitor concentration. From Fig. 7, the total impedance of mild steel in the presence of 0.5 mM decreases with increasing solution temperature. This behavior is due to desorption of adsorbed inhibitor molecules from the mild steel surface. In the impedance spectrum of mild steel in the presence of response, the Nyquist plots have two loops: one loop in the high frequency region (HF) and another at an intermediate frequency (MF), with slight inductive behavior at low frequencies (LF)The HF and MF loops are attributed to the electrode and charge-transfer process, respectively. The inductive behavior observed in the LF region is attributed to relaxation of the adsorption of corrosion products or to the adsorption of inhibitor molecules on the mild steel surface in acidic solution in the absence and presence of inhibitor, respectively^40^,^41^. Similar behavior is observed for all temperatures examined. The corroding surface of the working electrode is expected to be inhomogeneous due to its roughness; therefore, the capacitance is presented through a constant phase element (CPE). The EIS results were analyzed using the equivalent circuit mentioned elsewhere^42^, as shown in Fig. 8. The inhibition efficiencies (IE%) were calculated from the charge transfer resistance using the equation 1:


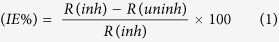


where *R*^o^_ct_ and *R*_ct_ indicate the charge transfer resistances in the presence and absence of corrosion inhibitor, respectively.

By using the software Gamry Analyst, EIS experimental data can be analyzed which were data matching CPE for mild steel/sample calculation solution resistance R_s_ and constant phase element CPE, calculation of charge transfer resistance R_ct_, and double layer charge, C_dl_^15^. Table 1, shows the EIS data for 1.0 M HCl with different corrosion inhibitor concentration at 30 °C, where the Cdl value decrease and the R_ct_ value increase with the increasing of inhibitor concentrations. This is due to the gradual replacement of water molecules by the adsorption of the inhibitor molecules on the metal surface, and decreasing the extent of dissolution reaction. The high Rct value are generally associated with slower corroding system^43^,^44^. While the decrease in Cdl value resulted from the decrease of the local dielectric constant and/or from the increase of thickness of the electrical double layer^45^. This proves that the corrosion inhibitor molecules absorbed on the surface of mild steel samples thus form a protective layer on the mild steel. Great resistance transfer charges commensurate with systems that corrode slowly^46^. With the increase in the value of R_ct_, the efficiency and capacity of inhibition (IE) also increased to 90.78% at a concentration of 0.5 mM.

According to Table 2, the rate of temperature increase from 30 °C to 60 °C has led to the R_ct_ and inhibition efficiency (IE) is decreasing. This is because the corrosion inhibitor molecules are adsorbed on the surface of the sample mild steel will undergo condensation when the temperature is increasing^47^. Adsorption of organic compounds can be divided into two main types, namely physical adsorption and interaction ‘chemisorption’. The presence of a transition metal, the void area, the low energy of the electron orbit, and the molecule inhibitors that have relatively loosely bound electrons which are very important in the process of corrosion inhibition^48^.

As shown in Tables 1 and 2, the Rct values increase with increasing concentration but decrease significantly with increasing solution temperature.

**Figure 9:**
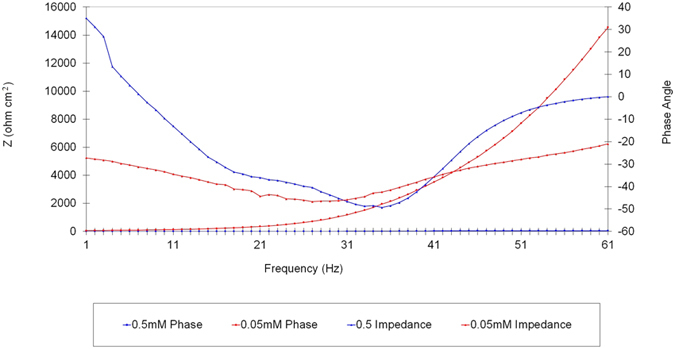
Experimental impedance and phase data in Bode format for mild steel in 1.0 M HCl containing 0.5 mM corrosion inhibitor denotes the fitted line using the equivalent circuit.

The next experiments is using the same concentration of corrosion inhibitor that is 0.5 mM but at a different temperatures which were 30 °C, 40 °C, 50 °C and 60 °C. Based on Fig. 7 we can see that the graph of semi-circle at 60 °C is the smallest and at 30 °C was greatest. This shows that the diameter of the semi-circle is getting smaller with increasing temperature. In other words, higher temperature, the smaller diameter of the semi-circle. These results suggest that the inhibition of corrosion is significantly reduced with increasing temperature. Increasing the temperature of the solution accelerates the corrosion process that occurs due to changes in the operating mechanism of corrosion^49^. In this work the filmed equivalent circuit model is used to describe the inhibitor-covered metal/solution interface. The circuit model is shown in Fig. 8, one time constant in Bode-phase has been identified from Fig. 9, Impedance and phase data in the form of Bode plots are illustrated in Fig. 9 for the mild steel aerated 1 M HCl solution at 30 °C and shows the line fit (fitted line) using the equivalent circuit model.

Different corrosion system (e.g., charge transfer control, diffusion control or a mixture type) may show different features in the EIS spectra. Through analyzing the EIS data (Nyquist plot, Bode plot), the corrosion mechanism of the system can be identified. In practice, EIS data are often interpreted in terms of electrical equivalent circuits that can be used to describe the electrical features of the electrochemical interfaces^50^. Most of the impedance spectra obtained for the corrosion of mild steel in HCl solutions consists of one depressed capacitive loop (one time-constant in Bode-phase representation) that corresponds to a charge-transfer process. The Bode plots of mild steel in 1 M HCl with and without the corrosion inhibitor as shown in Fig. 9. When the Nyquist plot contains a “depressed” semicircle with the center under the real axis, such behavior characteristic for solid electrodes and often referred to as frequency dispersion, which have been attributed to roughness and inhomogeneity of the surface^51^. Two ways are used in the literature to describe the EIS spectra for the inhomogeneous films on the metal surface or rough and porous electrodes. One is the finite transmission line model^52^ and the other is the filmed equivalent circuit model^53^, which is usually proposed to study the degradation of coated metals^54^. It has been suggested that the EIS spectra for the metal covered by organic inhibitor films are very similar to the failed coating metals^55^.

### Polarization measurements

**Figure 10:**
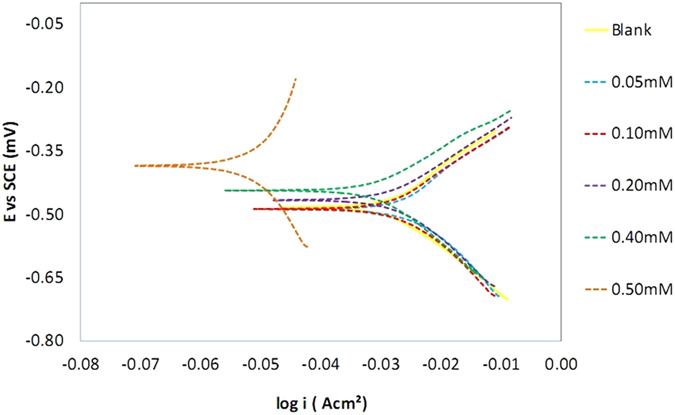
Potentiodynamic polarization curves for mild steel in 1.0 M HCl with various concentrations of azelaic acid dihydrazide at 30 °C.

**Figure 11:**
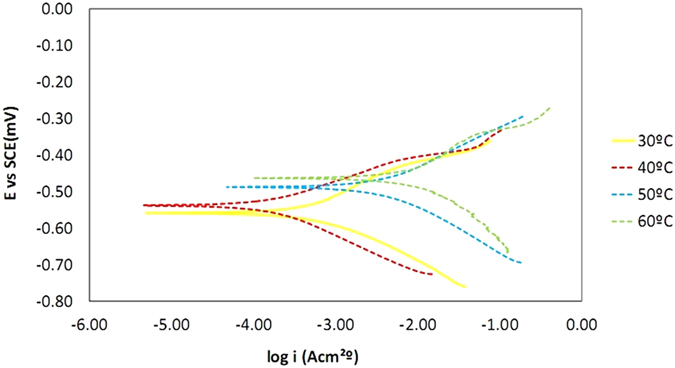
Potentiodynamic polarization curves for mild steel in 1.0 M HCl with 0.5 mM corrosion inhibitor at different temperatures.

The polarization profile of mild steel in 1.0 M HCl is shown in Figs 10 and 11. The numerical values of the variation of corrosion current density *(i*corr), corrosion potential (*E*_CORR_), anodic Tafel slope (βa) and cathodic Tafel slope (βc) with various concentrations of the azelaic acid dihydrazide inhibitor and at various solution temperatures were obtained from polarization profiles and are presented in Tables 3 and 4.

These values were calculated from the intersection of the anodic and cathodic Tafel lines of the polarization curve at ECORR. The inhibition efficiency (IE) was calculated using the equation 2:


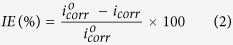


where 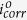 and 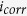 are the corrosion current densities in the absence and presence of the inhibitor, respectively.

Tables 3 and 4 show that 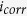 increases with increasing solution temperature, whereas 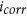 decreases with the addition of synthesized inhibitor to the acidic solution throughout the investigated temperature range. This behavior can be explained as follows: the inhibitor adsorbs onto the metal surface, and an increase in temperature results in the desorption of some adsorbed inhibitor molecules, exposing more metal surface to the acidic medium and thus increasing the metal dissolution rate and decreasing inhibition efficiency^56^. The addition of azelaic acid dihydrazide as an inhibitor causes the calculated E_CORR_ values to shift towards more positive values, which reflects the inhibitory effect of azelaic acid dihydrazide on the corrosion of mild steel at 30 °C; however, this value decreases with solution temperature, which indicates a decrease in the level of protection of azelaic acid dihydrazide. Figures 10 and 11 reveal that the anodic and cathodic processes change with the addition of various concentrations of azelaic acid dihydrazide. A compound can be classified as an anodic- or cathodic-type inhibitor when the change in E_CORR_ is greater than 85 mV^57^. Because its largest displacement is 385 mV at 30 °C (Table 3), therefore, azelaic acid dihydrazide functions as a mixed-type inhibitor, which indicates that the addition of azelaic acid dihydrazide to acidic solution reduces the anodic dissolution of mild steel and retards cathodic hydrogen.

### Electrochemical frequency modulation (EFM)

Electrochemical frequency modulation (EFM) is a new electrochemical technique for determining corrosion rate without preliminary knowledge of the Tafel constants. One principal advantage of this technique is that corrosion rate, Tafel parameters and causality factors are measured in a single data set^58^. While using EFM, a potential perturbation signal composed of two sine waves is applied to any corroding specimen to obtain a current response. EFM has been used for different combinations of metals and electrolytes to accurately measure corrosion parameters. This technique is similar to the harmonic method in that it employs a lower-amplitude (20 mV) sinusoidal perturbation signal but, unlike the harmonic method, is composed of two sine waves instead of one. EFM has many advantages over the harmonic method, including data validations, a larger current response and an insensitivity to harmonics in the perturbation signal. The corrosion parameters, including corrosion efficiency *IE* (%), corrosion current density (μA·cm−2), Tafel constant and causality factors (CF-2 and CF-3), are listed in Tables 5 and 6 for different concentrations of azelaic acid dihydrazide in 1.0 M HCl at 30 °C and at different temperatures, respectively.

As shown in Table 5, *i*corr decreases with increasing inhibitor concentration. The standard values for CF-2 and CF-3 are 2.0 and 3.0, respectively. If causality factors differ from 2 to 3, one may conclude that the measurement is affected by noise. If the value of the causality factor approximates the standards, a correlation exists between the perturbation and response signals; therefore, the data are accepted. If CF-2 and CF-3 are in the range of 0–2 and 0–3, the EFM data are valid. Any deviation in the causality factor from the theoretical value may be due to a perturbation amplitude that is too small, in insufficient resolution in the spectrum frequency, or an inhibitor that is not functioning properly^59^. As observed before with other measurements, the inhibition efficiency of azelaic acid dihydrazide increases with increasing inhibitor concentration but decreases with solution temperature at a given concentration (Table 6). This result suggests that the inhibitor molecules adsorb physically on the mild steel surface and not chemically; therefore, increasing temperature enhances both the dissolution of metal and the desorption of inhibitor molecules from the metal surface.

**Figure 12:**
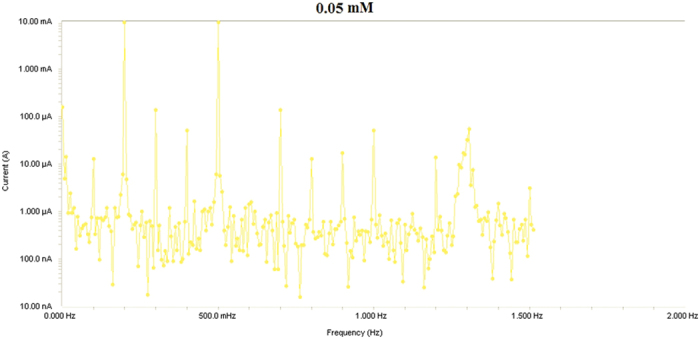
Intermodulation spectrum of mild steel in 1 M HCl in presence of 0.05 mM the corrosion inhibitor at 30 °C.

**Figure 13:**
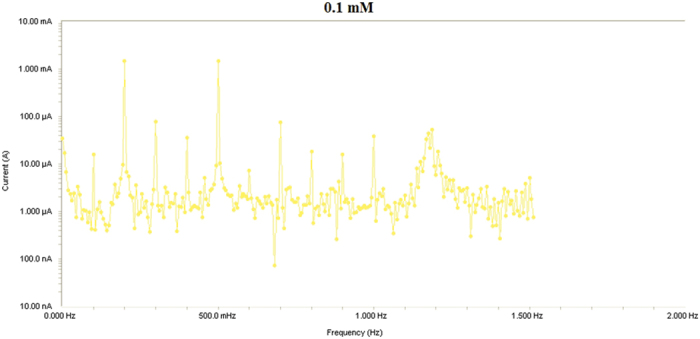
Intermodulation spectrum of mild steel in 1 M HCl in presence of 0.1 mM the corrosion inhibitor at 30 °C.

**Figure 14:**
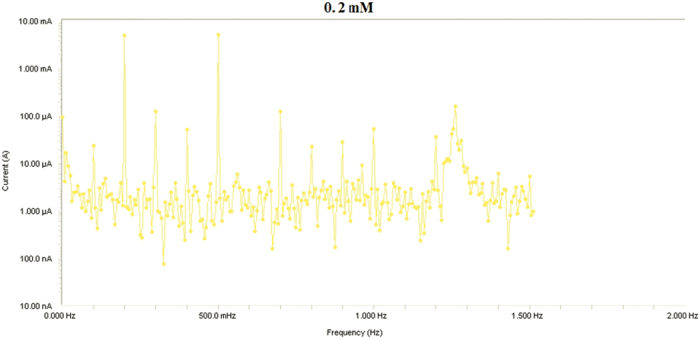
Intermodulation spectrum of mild steel in 1 M HCl in presence of 0.2 mM the corrosion inhibitor at 30 °C.

**Figure 15:**
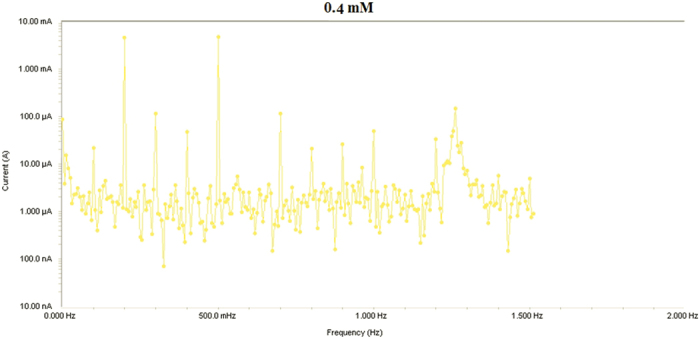
Intermodulation spectrum of mild steel in 1 M HCl in presence of 0.4 mM the corrosion inhibitor at 30 °C.

**Figure 16:**
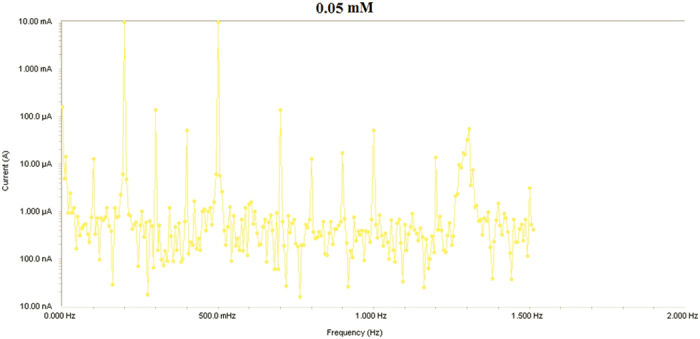
Intermodulation spectrum of mild steel in 1 M HCl in presence of 0.5 mM the corrosion inhibitor at 30 °C.

Results from Electrochemical frequency modulation (EFM) experiments are a spectrum of current response as function of frequency. The spectrum is called the intermodulation spectrum. Figures 12, 13, 14, 15, 16 represent the Electrochemical frequency modulation intermodulation spectra of mild steel in I M HCl in the absence and presence of different concentrations of the corrosion inhibitor at 30 °C.

### Postulated mechanism of inhibition

**Figure 17:**
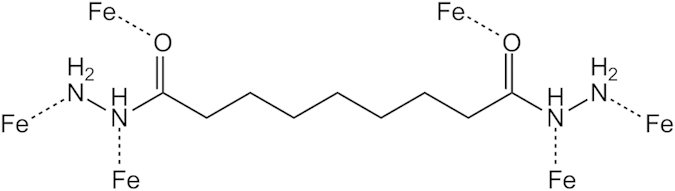
Mechanism of inhibition.

Inhibitors that are derived from organic molecules are adsorbed on the metal surface and prevent further dissolution of metal by blocking either the cathodic or anodic reaction or both. Organic inhibitors, capable of forming insoluble complexes, or chelates, with metallic ions are present on the surface of metal^44^. The inhibition efficiency of our corrosion inhibitor against the corrosion of steel in 1.0 M HCl can be explained on the basis of the number of adsorption sites, their charge density, molecular size, mode of interaction with the metal surface and ability to form a metallic complex. The π and free electrons on nitrogen and oxygen atoms form bonds with the metal surface, as in Fig. 17.

The inhibition efficiency of azelaic acid dihydrazide also can be explained according to valence Bond Theory(VBT), Crystal Field Theory (CFT) or molecular orbital theory (MOT) of complexation. Complexation between metal and ligand (azelaic acid dihydrazide) should done through coordination bonds that formed by transfer the ion pair from the nitrogen atoms of azelaic acid dihydrazide to d-orbital of metal.

### Scanning electron microscopy

**Figure 18:**
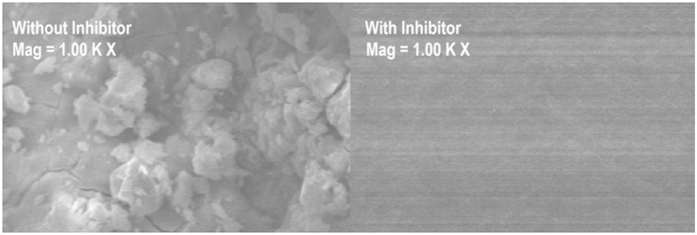
SEM micrographs of mild steel in 1.0 M HCl solution at 30 °C.

SEM analysis was performed to investigate the surface morphology of mild steel after immersion in 1.0 M HCl in the absence and presence of 0.5 mM azelaic acid dihydrazide for 3 h at 30 °C (Fig. 18). A damaged surface is observed in the absence of azelaic acid dihydrazide due to the high dissolution rate of iron at this pH; however, a thin and uniform layer on the metal surface is observed in the presence of azelaic acid dihydrazide. The cracks in the film are due to surface dehydration because the surface was dried prior to SEM imaging. This result is evidence that azelaic acid dihydrazide can be adsorbed on the mild steel surface and insulate the surface from the acidic medium.

## Conclusions

In this study, azelaic acid dihydrazide was synthesized and characterized using various spectroscopic methods. Changes in electrochemical impedance spectroscopy (EIS) and potentiodynamic polarization measurements were used to study the corrosion inhibition of mild steel in 1.0 M HCl solutions at 30, 40, 50 and 60 °C, using azelaic acid dihydrazide as an inhibitor. Azelaic acid dihydrazide exhibited excellent inhibition performance as a mixed-type inhibitor. In general, the acidic corrosion of mild steel was reduced by addition of an appropriate inhibitor concentration. The inhibition efficiencies increased with inhibitor concentration but were reduced proportionally with temperature. The inhibition efficiencies obtained from EIS data were comparable with those obtained from polarization measurements in which the inhibited solution had higher values than the uninhibited solution. The inhibitor acts as an efficient corrosion inhibitor on a mild steel surface that obeys the Langmuir adsorption isotherm. SEM micrographs demonstrated that the inhibitor molecules form a protective film on the steel surface.0 M sulfuric acid and exhibits a maximum inhibition efficiency of 93.31%.

## Additional Information

**How to cite this article**: Al-Amiery, A. A. *et al.* Synthesis and characterization of a novel eco-friendly corrosion inhibition for mild steel in 1 M hydrochloric acid. *Sci. Rep.* **6**, 19890; doi: 10.1038/srep19890 (2016).

## Figures and Tables

**Table 1:** CPE data for mild steel in 1.0 M HCl with different corrosion inhibitor concentrations at 30 °C.

Concentration (mM)	R_s_ (ohm cm^2^)	R_ct_ (ohm cm^2^)	CPE_dl_		C_dl_ (μF cm^−2^)	IE (%)
			Y_o_ (μS s^α^cm^−2^)	α		
Blank	0.2537	0.0781	0.0009	0.9174	3.387	0.00
0.05	0.3595	0.7617	0.0051	0.7096	1.375	89.75
0.10	0.3567	0.7754	0.0038	0.7258	0.8209	89.93
0.40	0.3927	0.7856	0.0017	0.8006	0.3659	90.06
0.50	0.5489	0.8479	0.0004	0.8584	0.2662	90.78

**Table 2:** CPE data for mild steel in 1.0 M HCl with 0.5 mM corrosion inhibitor at different temperatures.

Temp. (°C)	Conc. (mM)	R_s_ (ohm cm^2^)	R_ct_ (ohm cm^2^)	CPE_dl_		C_dl_ (μF cm^−2^)	IE (%)
				Y_o_ (μS s^α^cm^−2^)	α		
30	Blank	0.2537	0.0781	924.6	0.9174	338.7	0.00
	0.5	0.5489	0.8479	435.4	0.8584	266.2	90.78
40	Blank	0.2478	0.2195	4526	0.9278	502.0	0.00
	0.5	0.2311	0.3521	500.2	0.8381	398.5	73.66
50	Blank	0.2305	0.1501	1634.01	0.7321	835.9	0.00
	0.5	0.2077	0.3378	507.5	0.8392	266.1	55.57
60	Blank	0.1836	0.1193	2172.87	0.8470	920.8	0.00
	0.5	0.1756	0.2490	451.4	0.8619	554.5	52.09

**Table 3:** Polarization parameters for mild steel in 1.0 M HCl with various concentrations of corrosion inhibitor.

	Potentiodynamic polarization parameters (PD)				
Conc. (mM)	β_a_(V dec^−1^)	β_c_(V dec^−1^)	I_corr_ (μA cm^−2^)	Corrosion rate (mpy)	IE (%)
Blank	0.1359	0.1315	667.00	7.5910	0.00
0.05	0.1289	0.1276	598.00	6.9640	10.34
0.10	0.1104	0.1217	407.00	4.7360	38.98
0.20	0.1012	0.1184	343.00	3.9950	48.58
0.40	0.0877	0.1138	177.00	2.0580	73.46
0.50	0.6004	0.4030	44.620	0.0537	93.31

**Table 4:** Polarization parameters for mild steel in 1.0 M HCl with 0.5 mM corrosion inhibitor at different temperatures.

Temp.°C	Conc. mM	Potentiodynamic polarization parameters (PD)					
		β_a_(V dec^−1^)	β_c_(V dec^−1^)	I_corr_ (μA cm^−2^)	-E_corr_ (mV vs. SCE)	Corrosion rate (mpy)	IE (%)
30	Blank	0.1359	0.1315	550.00	493.00	7.5910	0.00
	0.5	0.6004	0.4030	44.620	385.00	0.0537	93.31
40	Blank	0.7033	0.6775	667.00	655.00	76.6600	0.00
	0.5	0.0707	0.0963	135.00	538.00	13.6900	75.45
50	Blank	0.9722	0.7179	824.00	674.00	114.8000	0.00
	0.5	0.1201	0.1290	416.00	487.00	423.1000	49.51
60	Blank	4.0150	2.0030	1564.00	673.00	622.4000	0.00
	0.5	0.1424	0.1424	999.00	463.00	1017.0000	36.13

**Table 5:** Electrochemical frequency modulation (EFM) parameters for mild steel in 1.0 M HCl with various concentrations of azelaic acid dihydrazide at 30 °C.

Conc. (mM)	Icorr (mA cm^−2^)	β1 (mV dec^−1^)	β2 (mV dec^−1^)	C.R. (mpy)	IEEFM (%)	CF-2	CF-3
Blank	3.759	81.67	93.75	390.3	0.00	1.101	3.26
0.05	2.832	104.4	156.5	288.3	24.66	2.038	2.241
0.10	1.637	88.05	124.9	166.7	56.45	1.993	2.845
0.20	1.590	89.54	132.6	161.8	57.70	1.936	3.407
0.40	0.873	90.81	121.8	88.86	76.78	2.063	5.051
0.50	0.0096	104.3	111.1	31.12	99.70	1.664	3.672

**Table 6:** EFM parameters for mild steel in 1.0 M HCl with 0.5 mM azelaic acid dihydrazide at various temperatures.

T (°C)	Conc. (mM)	Icorr (mA cm^−2^)	β1 (mV dec^−1^)	β2 (mV dec^−1^)	C.R. (mpy)	IEEFM (%)	CF-2	CF-3
30	Blank	3.7590	81.67	93.75	390.3	0.00	1.101	3.26
	0.5	0.0096	104.3	111.1	31.12	99.70	1.664	3.672
40	Blank	6.8050	89.93	111.9	692.8	0.00	1.994	2.994
	0.05	1.666	86.37	107.7	169.40	75.52	2.009	4.634
50	Blank	19.14	93.68	109.6	1948	0.00	2.057	5.767
	0.5	3.552	86.85	123.3	361.1	61.44	1.948	3.307
60	Blank	56.74	119.6	146.5	5776	0.00	1.813	2.916
	0.5	10.85	144.0	192.5	1104	60.87	1.899	3.135
